# Time Exposure and Fat Mass Reduction Drive Trimethylamine N-Oxide Modulation During a Very-Low-Energy Ketogenic Therapy (VLEKT) in Women with Obesity

**DOI:** 10.3390/metabo16030150

**Published:** 2026-02-24

**Authors:** Giuseppe Annunziata, Ludovica Verde, Maria Maisto, Martina Galasso, Giulia De Alteriis, Vincenzo Piccolo, Gian Carlo Tenore, Silvia Savastano, Annamaria Colao, Giovanna Muscogiuri, Luigi Barrea

**Affiliations:** 1 Department for the Promotion of Human Sciences and Quality of Life, San Raffaele Open University, 00166 Rome, Italy; 2 Dipartimento di Psicologia e Scienze della Salute, Università Telematica Pegaso, 80143 Naples, Italy; 3 Centro Italiano per la cura e il Benessere del Paziente con Obesità (C.I.B.O), Unità di Endocrinologia, Diabetologia e Andrologia, Dipartimento di Medicina Clinica e Chirurgia, Università degli Studi di Napoli Federico II, 80131 Naples, Italy; 4 Division of Endocrinology, Department of Medicine, The University of Arizona College of Medicine, Tucson, AZ 85724, USA; 5 Department of Pharmacy, University of Naples Federico II, 80131 Naples, Italy; 6 Unità di Endocrinologia, Diabetologia ed Andrologia, Dipartimento di Medicina Clinica e Chirurgia, Università degli Studi di Napoli Federico II, 80131 Naples, Italy; 7 Cattedra Unesco “Educazione Alla Salute E Allo Sviluppo Sostenibile”, Università degli Studi di Napoli Federico II, 80131 Naples, Italy

**Keywords:** TMAO, VLEKT, obesity, fat mass reduction, body composition, gut microbiota, cardiovascular risk

## Abstract

**Background/Objectives**: Trimethylamine N-oxide (TMAO) is a gut microbiota-derived metabolite increasingly recognized as a pro-atherogenic factor and a biomarker of cardiometabolic risk. Dietary patterns and adiposity are key modulators of circulating TMAO levels; however, evidence on the impact of very-low-energy ketogenic therapy (VLEKT) on TMAO metabolism, particularly in women with obesity, remains limited. This study aimed to investigate the effects of VLEKT on circulating TMAO concentrations, with specific focus on treatment duration and body composition (BC) changes. **Methods**: This study included 43 adult women with obesity eligible for VLEKT based on meal replacements. Anthropometric measurements and BC were assessed using standardized protocols and bioelectrical impedance analysis at baseline and post-intervention. Serum TMAO concentrations were quantified by validated HPLC–ESI–MS/MS. **Results**: After VLEKT, participants exhibited significant reductions in body weight, BMI, waist girth, fat mass (FM), and circulating TMAO levels. Greater reductions in TMAO were observed in women with longer ketogenic exposure and more pronounced FM loss. Changes in TMAO levels correlated negatively with VLEKT duration and positively with FM variations. In multivariate models, treatment duration and FM reduction emerged as independent predictors of TMAO decrease. A Receiver Operating Characteristic (ROC) analysis identified a FM reduction ≥14.25% as the optimal threshold associated with clinically relevant TMAO lowering. **Conclusions**: VLEKT reduces circulating TMAO levels in women with obesity. This effect appears to be primarily driven by the duration of ketogenic exposure and the magnitude of FM loss, rather than total weight reduction alone, highlighting the relevance of BC-targeted interventions in modulating gut microbiota-derived cardiometabolic risk markers.

## 1. Introduction

Obesity represents a major global health burden and is consistently associated with an increased risk of cardiovascular disease (CVD), driven by a complex interplay between adiposity, chronic low-grade inflammation, oxidative stress, and metabolic dysfunction. In this context, trimethylamine-N-oxide (TMAO) has recently emerged as a clinically relevant biomarker and a novel proatherogenic factor, closely involved in the development and progression of atherosclerosis, independently of traditional cardiovascular risk factors [1,2,3,4,5,6,7]. This association has been robustly confirmed by clinical trials and observational studies linking elevated circulating TMAO levels to adverse cardiovascular outcomes [2,8]. A large body of evidence indicates that elevated TMAO levels are associated with an increased incidence of major adverse cardiovascular events, including myocardial infarction, stroke, heart failure and cardiovascular mortality [9,10]. Meta-analytic data demonstrate a positive, dose-dependent relationship between circulating TMAO concentrations and cardiovascular events and mortality [11], while several studies suggest that TMAO predicts cardiovascular risk independently of classical risk factors, particularly in high-risk populations [12]. Accordingly, TMAO has been proposed as a prognostic biomarker for CVD beyond traditional risk stratification tools [12,13].

TMAO is a bioactive gut microbiota-derived metabolite originating from the metabolism of dietary precursors, mainly choline, betaine, phosphatidylcholine and L-carnitine, which are abundant in animal-derived foods such as red meat, eggs, dairy products, fish and seafood [1,2,14,15]. These substrates are converted by specific gut microbial strains expressing TMA lyases, carnitine oxygenases and related enzymes into trimethylamine (TMA), which is subsequently oxidized in the liver by flavin-containing monooxygenase 3 (FMO3) to form TMAO [1,2,15,16]. Importantly, alterations in gut microbiota composition significantly influence TMAO production in response to dietary patterns [17]. At the mechanistic level, TMAO has been implicated in key pathways linking obesity to cardiovascular risk. TMAO is a highly reactive molecule that contributes to oxidative stress, defined as an imbalance between the production and clearance of reactive oxygen species (ROS) [18,19]. Obesity and related metabolic disorders are characterized by increased ROS generation [20,21], which synergizes with TMAO-mediated effects on endothelial dysfunction, foam cell formation, plaque instability and platelet activation, thereby accelerating atherogenesis [1,22,23]. Furthermore, TMAO promotes vascular inflammation by enhancing leukocyte recruitment, upregulating adhesion molecules and pro-inflammatory cytokines, and activating inflammatory pathways such as the NLRP3 inflammasome [24,25].

Given its gut-derived origin, dietary modulation represents a primary strategy for controlling circulating TMAO levels. Diets rich in fiber and antioxidants, such as the Mediterranean diet, have been proposed as beneficial approaches to reduce TMAO production through microbiota modulation [26,27,28]. However, while such dietary patterns are associated with cardiovascular benefits, their effectiveness in inducing substantial weight loss and fat mass (FM) reduction in individuals with obesity is often limited, particularly in the short-to-medium term. This limitation is clinically relevant, as excess adiposity itself contributes to elevated oxidative stress, inflammation and cardiometabolic risk, which may sustain high TMAO levels despite qualitative dietary improvements. Similarly, although weight loss interventions are generally associated with improvements in cardiometabolic risk, evidence regarding their effects on circulating TMAO levels remains inconsistent. In particular, a diet-induced weight loss study reported that decreases in circulating TMAO were not consistently associated with improvements in bone health and were influenced by dietary fat intake and changes in L-carnitine, a key TMAO precursor [29]. These findings suggest that reductions in TMAO during weight loss may reflect complex metabolic adaptations rather than a direct consequence of weight loss itself. On the other hands, short-term lifestyle interventions appear insufficient to induce robust TMAO reductions in women with obesity. Indeed, neither a low-calorie diet (LCD) nor an LCD combined with interval training for two weeks significantly reduced plasma TMAO levels overall, although reductions were observed in individuals with higher baseline TMAO concentrations [30]. Notably, these interventions lowered choline, TMA and carnitine concentrations, which were associated with improvements in central hemodynamics, despite the absence of a consistent reduction in TMAO itself [30]. Together, these findings suggest that modest or short-term weight loss may be inadequate to substantially modify TMAO metabolism, highlighting the need to explore the role of more pronounced metabolic and body composition (BC) changes. Conversely, recent evidence indicates that longer hypocaloric interventions may reduce circulating TMAO levels, yet through mechanisms not directly attributable to changes in dietary precursor intake or major shifts in gut microbiome diversity [31]. This suggests that factors beyond diet composition alone—potentially including changes in BC—may contribute to TMAO modulation during sustained weight loss. In particular, it is still unclear whether TMAO reductions observed during weight loss are driven by body weight loss per se or by the selective reduction of FM, which plays a central role in obesity-related inflammation and oxidative stress.

In this context, ketogenic dietary interventions have gained increasing attention as effective non-pharmacological strategies for obesity management. The very-low-energy ketogenic therapy (VLEKT), formerly referred to as very-low-calorie ketogenic diet, is characterized by a carbohydrate intake below 30–50 g/day and an energy intake not exceeding 800 kcal/day [32]. VLEKT induces nutritional ketosis, a physiological state distinct from diabetic ketoacidosis, and promotes marked reductions in body weight and FM through hormonal and metabolic adaptations, including reduced insulin secretion, enhanced lipolysis and increased ketone body utilization [33,34,35]. Beyond weight loss, VLEKT has been shown to improve glycemic control, reduce systemic inflammation and oxidative stress, and modulate neuroendocrine pathways [36]. By inducing rapid and marked reductions in FM, together with improvements in inflammation and oxidative stress, VLEKT represents a valuable model to investigate whether FM reduction is the primary driver of TMAO reduction during weight loss, beyond changes in body weight or dietary precursor intake alone.

However, it is noteworthy that classical ketogenic diets based on fresh foods often include a high intake of animal-derived proteins and fats. Given that foods rich in choline and L-carnitine represent major dietary sources of TMAO precursors [2,15,37,38,39,40], such dietary patterns may theoretically promote increased TMAO production, potentially counteracting some of the cardiovascular benefits of weight loss. This aspect represents a critical, yet underexplored, issue in the context of ketogenic interventions and TMAO modulation.

An alternative approach is represented by VLEKT protocols based on the structured use of replacement meals, which allow precise control of macronutrient composition and caloric intake while maintaining nutritional ketosis [41]. The use of replacement meals may limit excessive intake of animal-derived proteins and TMAO precursors, while preserving the metabolic advantages of ketosis and substantial FM reduction. Moreover, emerging evidence suggests that VLEKT may favorably influence gut microbiota composition and intestinal dysbiosis, further contributing to the modulation of microbiota-derived metabolites implicated in cardiometabolic risk [36].

Taken together, these considerations support the hypothesis that, in women with obesity, TMAO modulation during VLEKT may be driven not only by changes in dietary precursor intake and microbiota composition, but also by the magnitude of FM reduction and the specific dietary implementation, particularly the use of replacement meals. Investigating these mechanisms may provide novel insights into the cardiometabolic effects of ketogenic therapies and the role of TMAO as a modifiable metabolic mediator.

The primary aim of this study was to investigate the effects of a VLEKT on circulating TMAO levels, with particular focus on the role of treatment duration and body composition (BC) changes in a cohort of adult women with obesity.

## 2. Materials and Methods

### 2.1. Design and Setting

This study was conducted on adult women with obesity eligible for a VLEKT protocol for weight loss at the *Centro Italiano per la cura e il Benessere del paziente con Obesità* (C.I.B.O) of Federico II University Hospital (Naples). Data were retrospectively collected between September and December 2025. After being informed about the study protocol, all women provided written consent. The Local Ethical Committee approved the study design (reference n. 50/20).

### 2.2. Study Population

In this study we enrolled a total of 65 women eligible for VLEKT protocol. To enhance sample homogeneity, only adult (>18 years), non-smoking women with obesity and no regular physical activity (less than 30 min of aerobic exercise *per* day), eligible for VLEKT protocol according to the European Association for the Study of Obesity (EASO) guidelines [33] were included. Exclusion criteria were:

- Age < 18 years
- Body mass index (BMI) < 30.0 kg/m^2^
- Presence of one or more absolute contraindications for VLEKT
- Women with diagnosis of gastrointestinal disorders
- Women who are currently pregnant or who have been breastfeeding within the preceding six months
- Women reporting a recent and clinically relevant body weight variation, defined as a change exceeding 10% within the previous six months
- Presence of endocrine conditions known to influence BC or nutritional status, including biochemical and/or clinical hyperandrogenism, oligo-ovulation or oligo-amenorrhea associated with polycystic ovary syndrome, as well as secondary endocrine causes, in accordance with Endocrine Society criteria [42]
- Chronic pathological conditions associated with impaired fluid balance, such as chronic hepatic or renal disorders, malignant diseases, and acute or persistent inflammatory states
- Current or recent use of pharmacological agents known to affect BC, nutrient metabolism, or body weight regulation
- Recent exposure to antibiotic therapies
- Adherence, within the preceding three months, to specific dietary interventions, including ketogenic, vegan, or vegetarian regimens, as well as the intake of antioxidant, vitamin, or mineral supplements
- Presence of implanted cardiac devices, such as pacemakers or implantable cardioverter-defibrillators, due to the potential theoretical risk of interference with bioelectrical impedance analysis (BIA) measurements.

**Figure 1:**
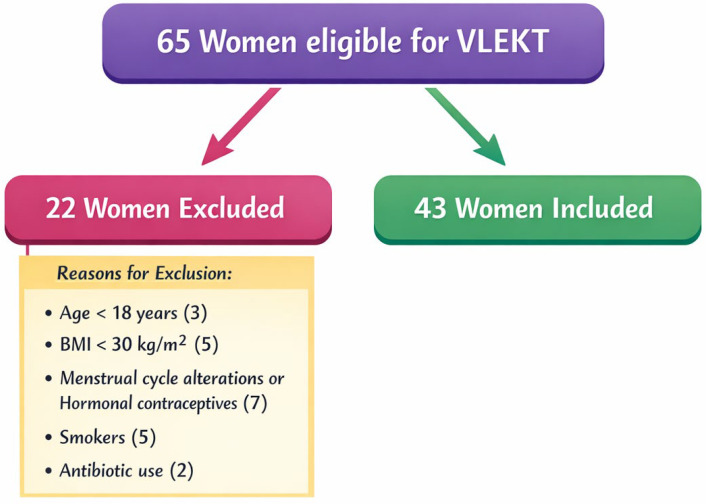
Study flowchart. Abbreviations: very-low-energy ketogenic therapy, VLEKT; body mass index, BMI.

Figure 1 shows a flow chart of included and excluded women.

### 2.3. Study Protocol

The study design comprised two main assessment points, conducted before and after the VLEKT intervention. At baseline, participants underwent an extensive clinical evaluation performed jointly by an Endocrinologist and a Nutritionist to determine eligibility. Subjects fulfilling all predefined inclusion and exclusion criteria were subsequently enrolled and provided written informed consent. During this initial phase, the Endocrinologist performed a comprehensive medical examination to confirm study eligibility. Thereafter, the Nutritionist evaluated nutritional status through anthropometric measurements and BC analysis and formulated an individualized VLEKT dietary plan. Participants received tailored counseling aimed at optimizing compliance with the prescribed nutritional regimen. Concurrently, with assistance from trained nursing personnel, venous blood samples were obtained for the determination of circulating TMAO concentrations. Participants were additionally instructed to maintain their habitual lifestyle behaviors throughout the intervention period.

During the VLEKT intervention, scheduled follow-up assessments were conducted via weekly telephone interviews administered by the Nutritionist to monitor dietary adherence and ketosis status. Compliance with the dietary protocol was evaluated by verifying the consumption of the prescribed number of VLEKT meal replacements, daily fluid intake of at least 2 L, and adherence to the written dietary instructions. Ketosis status was assessed by measuring capillary blood ketone levels; for study purposes, ketosis was recorded dichotomously as present or absent (YES/NO). At each follow-up contact, the Nutritionist also documented any deviations from the protocol, including changes in physical activity or variations in food and beverage intake.

At the conclusion of the intervention, participants underwent a final endocrinological and nutritional evaluation. Blood sampling was repeated to allow for post-intervention analysis of circulating TMAO levels.

The duration of the active ketogenic phase was individualized according to clinical practice and patient preference, reflecting real-world VLEKT implementation. Specifically, the length of the intervention was determined jointly by the Endocrinologist and the Nutritionist based on clinical response, achievement of initial weight-loss targets, and patient willingness to continue the ketogenic phase. Consequently, the duration of ketogenic exposure varied among participants. In the present cohort, the mean duration of the active phase ranged approximately from 28 to 45 days. Anthropometric measurements, body composition assessment, and blood sampling for circulating TMAO determination were performed at two predefined time points: immediately before the initiation of the active ketogenic phase (baseline) and at the end of the active ketogenic phase (post-intervention), prior to the transition to the subsequent nutritional re-education phase. Due to the relatively limited sample size, separate statistical analyses according to different intervention durations were not feasible. Therefore, treatment duration was analyzed as a continuous variable in correlation and regression models.

**Figure 2:**
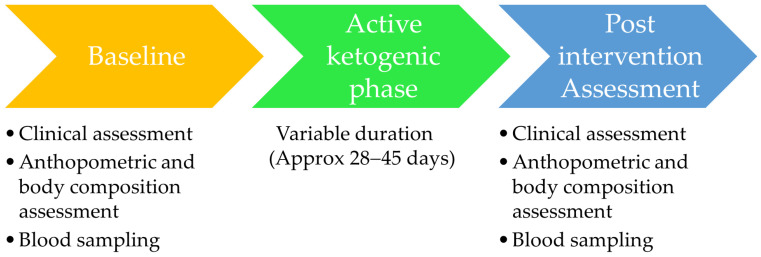
Overview of the very-low-energy ketogenic therapy (VLEKT) intervention protocol and assessment timepoints.

A schematic representation of the intervention phases and assessment time points is shown in Figure 2.

### 2.4. Anthropometric Measurements and Body Composition Assessment

Anthropometric measurements were performed by a certified clinical nutrition specialist in strict accordance with the International Society for the Advancement of Kinanthropometry (ISAK, 2006) standards [43]. All assessments were conducted in the morning hours, between 08:00 and 10:00 a.m., after an overnight fast. Participants were evaluated barefoot and wearing light clothing. Body weight was measured using a calibrated mechanical balance scale (Seca 711; Seca, Hamburg, Germany), while stature was determined with a wall-mounted stadiometer from the same manufacturer (Seca 711; Seca, Hamburg, Germany). BMI was subsequently derived as body weight (kg) divided by height squared (m^2^). Based on World Health Organization (WHO) classification criteria, BMI values between 30.0 and 34.9 kg/m^2^ were indicative of class I obesity, values between 35.0 and 39.9 kg/m^2^ indicative of class II obesity, and ≥40.0 kg/m^2^ indicative of class III obesity [44]. Waist girth (WG) was assessed according to the National Center for Health Statistics protocol using a non-elastic measuring tape. Measurements were taken at the level of the natural waist indentation; in its absence, the midpoint between the inferior margin of the rib cage and the iliac crest was used as the anatomical reference. All values were recorded with a precision of 0.1 cm.

BIA was conducted using a phase-sensitive analyzer operating at a frequency of 50 kHz and a current of 800 µA (BIA 101 BIVA; Akern Ltd., Pisa, Italy), in compliance with the European Society for Clinical Nutrition and Metabolism (ESPEN) recommendations [45]. Prior to each assessment session, the instrument was calibrated using resistors and capacitors of known electrical properties. Measurements were obtained in fasting conditions, with participants instructed to refrain from vigorous physical activity for at least six hours and from alcohol consumption for 24 h prior to evaluation. Subjects were positioned supine, with upper and lower limbs slightly abducted from the trunk. After skin cleansing with alcohol, disposable electrodes (BIATRODES; Akern Srl, Florence, Italy) were applied to the dorsal surfaces of the right hand and foot. On the hand, electrodes were placed at the metacarpophalangeal joint and midway between the distal prominences of the radius and ulna; on the foot, electrode placement corresponded to the transverse arch and the midpoint between the medial and lateral malleoli. Electrical resistance (Rz) and reactance (Xc) were directly measured, and phase angle (PhA) was calculated as the arctangent of the ratio Xc/Rz, expressed in degrees (PhA = arctan[Xc/R] × 180/π). Among BC–related variables, FM and skeletal muscle mass (SMM), expressed in kilograms and percentage were considered. SMM was estimated using the Janssen predictive equation:

SMM = (height^2^/R × 0.401) + (sex × 3.825) + (age × −0.071) + 5.102,

where height is expressed in centimeters and sex is coded as 0 for females and 1 for males [46]. Hydration-related parameters, including total body water (TBW), intracellular water (ICW), and extracellular water (ECW), were quantified and reported both as absolute values (liters) and relative percentages. All bioimpedance-derived variables were computed using dedicated software (Bodygram Plus; Akern, Florence, Italy).

### 2.5. Determination of Circulating TMAO Levels

Venous blood specimens were obtained from all participants during the morning hours following an overnight fasting period, using serum separator tubes containing a clot activator and gel matrix (BD Vacutainer). After collection, samples were centrifuged at 1700× *g* for 20 min, and the resulting serum was subdivided into aliquots and preserved at −80 °C until further biochemical analyses were performed.

Serum concentrations of TMAO were quantified by high-performance liquid chromatography coupled to tandem mass spectrometry with heated electrospray ionization (HPLC–ESI–MS/MS), following serum protein removal by methanolic precipitation. Quantitative determination was achieved using an external calibration approach, and the analytical procedure was fully validated in terms of linearity, sensitivity, precision, and accuracy in compliance with internationally recognized regulatory standards.

For sample preparation, serum proteins were precipitated using ice-cold methanol. Briefly, 50 μL of serum were aliquoted into 0.5 mL microcentrifuge tubes, followed by the addition of 175 μL of chilled methanol. After vortex mixing for 10 s, samples were incubated on ice for 40 min to ensure complete protein precipitation. The mixtures were subsequently centrifuged at 14,000× *g* for 10 min at 4 °C. The resulting clear supernatants (approximately 120 μL) were carefully transferred into amber autosampler vials equipped with inserts and subjected to LC–MS/MS analysis.

Chromatographic separation and quantification of serum TMAO were carried out using a DIONEX UltiMate 3000 HPLC system (Thermo Fisher Scientific, San Jose, CA, USA), equipped with an autosampler and a binary solvent delivery module, coupled to an LTQ XL triple quadrupole mass spectrometer (Thermo Fisher Scientific, San Jose, CA, USA). Separation was achieved on a hydrophilic interaction liquid chromatography (HILIC) column (Luna HILIC, 50 × 2.1 mm, 1.7 μm particle size), protected by a matching HILIC guard column (Phenomenex, Torrance, CA, USA). The mobile phase consisted of solvent A (water containing 0.15% formic acid and 10 mM ammonium acetate) and solvent B (LC–MS grade methanol). Isocratic elution was performed using an 80:20 (A:B) ratio over a 6-min run. The column temperature was maintained at 60 °C, the injection volume was set at 5 μL, and the flow rate was fixed at 0.35 mL/min.

Mass spectrometric detection was performed using a heated electrospray ionization source operating in positive ion mode, with analyte detection achieved via multiple reaction monitoring (MRM). Argon was employed as the collision gas at a collision energy of 35.0 eV. The selected quantifier and qualifier ion transitions for TMAO were *m*/*z* 76.1 → 59.1 and *m*/*z* 76.1 → 58.1, respectively. MRM conditions were optimized using a 1 ppm methanolic TMAO standard solution.

Quantification was based on an external calibration curve exhibiting excellent linearity (R^2^ ≥ 0.99), generated from nine calibration levels (2500, 1250, 625, 312.5, 156.2, 78.1, 39, 19.5, and 9.8 ppb). Calibration standards were prepared through serial 1:2 dilutions and analyzed in triplicate. The ESI source parameters were configured as follows: sheath gas flow rate, 38; auxiliary gas flow rate, 8; capillary temperature, 320 °C; vaporizer temperature, 130 °C; spray voltage, 3.5 kV; source current, 100 μA; capillary voltage, 29 V; and tube lens voltage, 60 V.

The HPLC–ESI–MS/MS method underwent comprehensive validation in accordance with International Council for Harmonization (ICH) guidelines [47], encompassing assessments of linearity, limits of detection (LOD) and quantification (LOQ), intra- and inter-day precision, accuracy, analytical stability, and carry-over effects. The analytical stability was assessed during the analytical run by evaluating the reproducibility of the peak area of a quality control (QC) sample obtained by pooling aliquots from all serum specimens. The details about the analytical validation were previously reported by Annunziata et al. (2025) [48].

### 2.6. VLEKT Intervention

In accordance with the European Association for the Study of Obesity (EASO) recommendations [33] and the consensus document issued by the working group of the Club of the Italian Society of Endocrinology (SIE) on dietary therapies in endocrinology and metabolism [34], the VLEKT is structured into sequential phases, namely an initial active (ketogenic) phase, followed by a nutritional re-education (non-ketogenic) phase, and a subsequent maintenance phase. The present investigation focused exclusively on the active ketogenic phase of the protocol.

During this phase, the dietary regimen was characterized by a markedly hypocaloric energy intake, set below 800 kcal per day, with a predefined macronutrient distribution. Specifically, carbohydrates accounted for approximately 13% of total energy intake, corresponding to less than 30 g/day; protein provided 43% of total energy, with an intake calibrated at 1.3 g per kilogram of ideal body weight; and fats contributed the remaining 44% of daily caloric intake. Ideal body weight (kg) was estimated using the Lorentz formula [49]:

ideal body weight = height (cm) − 100 − [(height − 150)/2].

Throughout the ketogenic phase, dietary intake relied predominantly on meal replacements formulated with proteins of high biological value. These protein sources included whey, soy, egg-derived proteins, and pea protein. To prevent micronutrient deficiencies and ensure overall nutritional adequacy during the intervention, a comprehensive supplementation regimen was systematically implemented. This supplementation comprised B-complex vitamins, vitamins C and E, essential electrolytes and minerals (potassium, sodium, magnesium, and calcium), as well as omega-3 polyunsaturated fatty acids.

The active phase of the VLEKT protocol was jointly designed by a qualified Nutritionist and formally approved by an Endocrinologist, ensuring both nutritional and clinical appropriateness.

According to the KeNuT multistep dietary model with meal replacements proposed by the SIE—Diet Therapies in Endocrinology and Metabolism working group [34], the daily meal structure during the active ketogenic phase followed a standardized scheme. Breakfast and snacks consisted exclusively of high–biological value protein meal replacements. Lunch and dinner also included protein meal replacements, complemented by low-glycemic index and low-glycemic load vegetables with minimal sugar content, together with the addition of one tablespoon of extra virgin olive oil per main meal. Mandatory micronutrient supplementation, including vitamins (B-complex, C, and E), minerals (potassium, sodium, magnesium, and calcium), and omega-3 fatty acids, was prescribed throughout the entire active phase.

In accordance with the individualized clinical approach adopted in routine practice, the duration of the active ketogenic phase was not fixed a priori but was tailored to each patient. The transition to the subsequent phases occurred once the predefined clinical targets were achieved or based on shared clinical decision-making. For the purpose of the present study, only the active ketogenic phase was considered, and all outcome measurements were performed immediately before its initiation and at its completion.

### 2.7. Statistical Analysis

Statistical analyses were performed using MedCalc^®^ software (version 12.3.0; MedCalc Software bvba, Mariakerke, Belgium) and IBM SPSS Statistics (PASW version 21.0; SPSS Inc., Chicago, IL, USA). The analytical dataset included exclusively female participants for whom complete measurements were available both at baseline and following completion of the active VLEKT phase. Continuous variables were reported as mean values with corresponding standard deviations (mean ± SD), while relative changes over time were expressed as percentage variations (Δ%). The normality of data distribution was evaluated using the Kolmogorov–Smirnov test. Differences between baseline values and post-intervention measurements were assessed using the paired Student’s *t*-test. Associations between baseline and post-VLEKT outcomes, expressed as percentage changes (Δ%), were examined using Spearman’s rank correlation coefficient. Variables demonstrating statistically significant correlations were subsequently entered into a stepwise multiple linear regression model, with percentage change in circulating TMAO levels (Δ%) specified as the dependent variable. Regression outputs were reported as standardized beta coefficients (β), t statistics, and coefficients of determination (R^2^). In addition, receiver operating characteristic (ROC) curve analysis was conducted to evaluate the discriminatory performance of FM variations in predicting circulating TMAO reductions exceeding the median value (−41.10%). The analysis yielded estimates of the area under the curve (AUC), optimal cut-off thresholds, sensitivity, specificity, standard error, and corresponding 95% confidence intervals (CIs).

## 3. Results

In this study, a total of 43 adult women (age on average 26.84 ± 8.74 years) with obesity were analyzed. Baseline characteristics of study participants are reported in Table 1. On average, before starting the VLEKT protocol study participants were with grade I obesity (mean BMI: 33.60 ± 3.54 kg/m^2^) with a mean WG of 101.07 ± 11.00 cm. BC analysis showed mean values of PhA of 5.80 ± 0.49°, FM of 44.05 ± 9.15 kg (47.33 ± 4.28%), and SMM of 26.29 ± 3.29 kg (28.74 ± 4.29%). Mean circulating TMAO levels were 5.11 ± 1.61 µM.

Variations of anthropometric measurements and BC parameters after the VLEKT protocol period are reported in Table 2. After the active phase of the VLEKT protocol, we observed significant reductions in body weight and BMI (−8.04 ± 1.63%, *p* < 0.001) and FM (kg: −17.34 ± 4.38%; %: −10.17 ± 3.39%, *p* < 0.001 for both). On the other hand, significant increases were observed in PhA values (10.54 ± 8.80%, *p* < 0.001) and %SMM (9.61 ± 4.30%, *p* < 0.001), while no significant differences were observed in SMM expressed in kg (*p* = 0.269).

**Figure 3:**
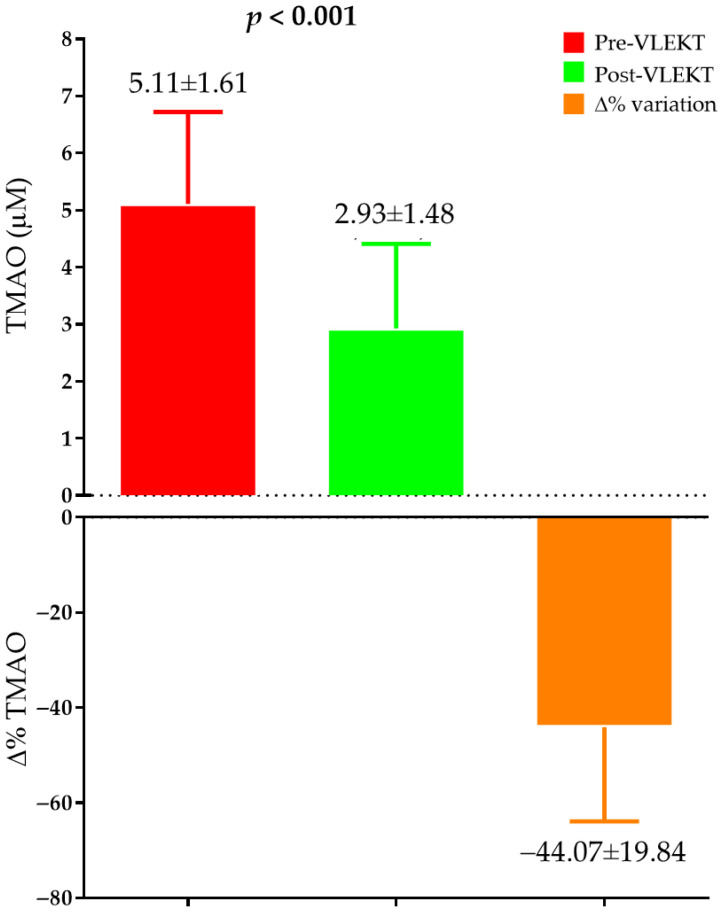
Pre and post VLEKT variations in TMAO serum levels. Data are presented as mean ± SD. Paired-sample Student’s *t*-test was used to compare values at baseline (pre-VLEKT, red column) and after the ketogenic protocol (post-VLEKT, green column). *p* < 0.05 was considered statistically significant. ∆% indicates the percent change from baseline to post-VLEKT (orange column). Abbreviations: TMAO, Trimethylamine-N-oxide.

Significant reductions in circulating levels of TMAO were observed after the VLEKT period, as shown in Figure 3.

Study participants were examined by stratifying above and below the median of variations (Δ%) in circulating TMAO levels (−41.10%) (Table 3). Women that were above the median of Δ%TMAO followed the VLEKT for a longer time (44.26 ± 3.54 days) than women below the median (35.65 ± 8.68 days). In addition, women that were above the median exhibited greater reductions in BMI (Δ% = −7.74 ± 1.71 vs. −8.55 ± 1.39, *p* = 0.028; above the median vs. below the median) and FM (Δ% FM (kg) = −18.70 ± 3.84 vs. −15.79 ± 4.52, *p* = 0.030; Δ% FM (%) = −11.14 ± 2.98 vs. −9.07 ± 3.56, *p* = 0.047; above the median vs. below the median), compared to women below the median.

Variations in circulating levels of TMAO after the VLEKT protocol (Δ%) were correlated with variations in duration of the ketogenic protocol, anthropometric measurements and BC parameters. As shown in Table 4, variations in TMAO levels correlated negatively with duration of VLEKT (r = −0.647, *p* < 0.001) and Δ%Xc (r = −0.375, *p* = 0.013) and positively with Δ% in weight and BMI (r = 0.403, *p* = 0.007), Δ%ECW (L; r = 0.389, *p* = 0.010), and FM (kg, r = 0.427, *p* = 0.004; %, r = 0.411, *p* = 0.006).

To assess the predictor value of variations in Xc, weight, BMI, ECW, and FM on variations in circulating levels of TMAO a multiple regression analysis was conducted with Δ%TMAO as dependent variable. As reported in Table 5, duration of VLEKT entered at the first step (*p* < 0.001), followed by Δ%FM and Δ%BMI.

**Figure 4:**
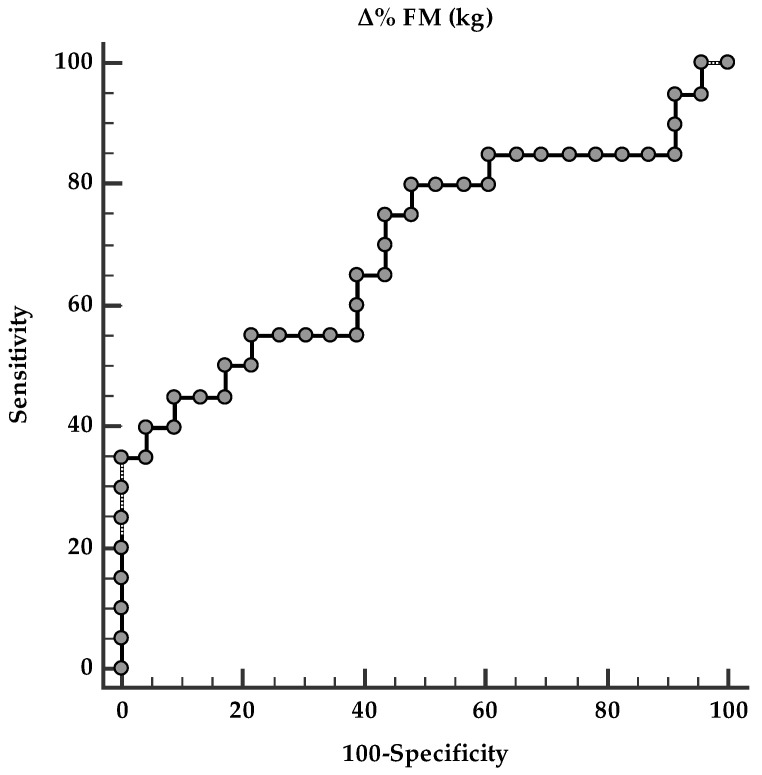
ROC for predictive fat mass variations in detecting changes in circulating TMAO levels.

A ROC curve analysis was performed to identify the optimal fat mass (FM, kg) cut-off value predictive of changes in circulating TMAO levels following VLEKT. The analysis identified an FM reduction ≥ 14.25% as the optimal threshold associated with the most pronounced changes in circulating TMAO levels after VLEKT (AUC = 0.698, standard error = 0.085, 95% confidence interval = 0.200–0.500; *p* < 0.001) (Figure 4).

## 4. Discussion

The present study demonstrates that adherence to a VLEKT is associated with a significant reduction in circulating TMAO levels in women with obesity, and provides novel evidence that this modulation is primarily driven by duration of ketogenic exposure and FM reduction, rather than by total body weight loss per se. These findings expand current knowledge on the metabolic effects of ketogenic nutritional therapies, highlighting BC and treatment duration as critical determinants of TMAO metabolism.

Previous studies have shown that VLEKT can induce significant metabolic improvements with emerging evidence of sex-specific responses [50,51,52]. However, data on TMAO modulation during VLEKT, particularly in women, remain scarce. To the best of our knowledge only two studies conducted on women with skin diseases observed significant reductions in TMAO serum levels after VLEKT as secondary outcome [53,54]. Our findings are in line with these previous reports and extend existing knowledge by demonstrating that reduction in cardiometabolic risk is closely linked to FM loss and ketogenic exposure duration rather than total weight loss. TMAO, indeed, is increasingly recognized as a pro-atherogenic metabolite, mechanistically linked to cardiometabolic risk, and its circulating concentrations are strongly influenced by dietary intake and gut microbiota metabolism [55].

It is well established that the consumption of foods rich in TMAO precursors—such as eggs, red meat, and fish—significantly increases plasma and urinary TMAO levels [17]. Tang et al. demonstrated that the generation of TMAO following a phosphatidylcholine challenge is strictly dependent on intestinal microbiota activity, as shown by the complete suppression of TMAO production after broad-spectrum antibiotic administration in healthy subjects [56]. Similarly, Miller et al. reported a clear dose–response relationship between egg consumption and both plasma and urinary TMAO levels, with substantial interindividual variability likely driven by differences in gut microbial composition and hepatic flavin-containing monooxygenase 1 (FMO1) and FMO3 activity [57]. In humans, fish consumption leads to a rapid and pronounced increase in circulating TMAO—approximately 50-fold higher than that observed after egg or beef intake—suggesting a direct absorption of free TMAO rather than microbial conversion from precursors [17]. However, disentangling the contribution of individual foods or nutrients to TMAO levels in free-living populations remains challenging due to dietary complexity and nutrient collinearity [58]. Nonetheless, high intake of eggs and meat, both rich in choline and carnitine, has been implicated in the proposed role of TMAO as a mechanistic link between diet, gut microbiota, and atherosclerosis development [40].

Within this framework, one of the most relevant findings of this study is the clear time-dependent reduction of TMAO concentrations, which became more pronounced with prolonged exposure to nutritional ketosis. This observation suggests that short-term ketogenic interventions may be insufficient to induce stable changes in TMAO metabolism, whereas sustained ketosis may progressively affect the host–microbiota–liver axis involved in TMAO production.

TMAO generation depends on the microbial conversion of dietary precursors (choline, phosphatidylcholine, carnitine) into TMA, followed by hepatic oxidation via FMO3 [55]. It is well accepted that prolonged carbohydrate restriction following ketogenic diets, may modulate gut microbiota mainly via decreasing alpha diversity and richness [59]. In particular, this seems to be primary due to the protein intake. The influence of dietary proteins on the gut microbiota warrants careful consideration, given the heterogeneous effects associated with different protein sources. Both the qualitative nature and the origin of dietary proteins represent critical determinants of their interaction with the intestinal microbial ecosystem [59]. A growing body of evidence has explored the extent to which various protein sources—specifically plant- versus animal-derived proteins—modulate gut microbiota composition and functionality [60,61,62]. Although high-protein dietary patterns have generally been associated with unfavorable alterations in gut microbial abundance and diversity [63], the overall impact on microbial ecology appears to be highly variable and dependent on protein type [64]. Experimental investigations have demonstrated that plant-based proteins exert more favorable modulatory effects on gut microbial profiles. For instance, mung bean protein administered within a high-fat dietary context was shown to increase the relative abundance of *Bacteroidetes* while concomitantly reducing *Firmicutes*. Similarly, pea protein supplementation promoted the proliferation of beneficial bacterial genera, including *Bifidobacterium* and *Lactobacillus* [60]. Collectively, these findings suggest that proteins of plant origin are associated with improvements in gut microbiota composition, accompanied by favorable metabolic effects in the host. In light of the available evidence, the preferential use of plant-derived protein sources during VLEKT is advisable, as these appear to support a more favorable gut microbial environment and may confer additional metabolic health benefits [59], including reductions in circulating gut microbiota-derived metabolites, such as TMAO.

In this sense, a key strength of this study the controlled nutritional protocol administered to study participants, formulated according the KeNuT guidelines for a VLEKT with replacement meals [34]. The use of structured meal replacements within the VLEKT protocol, indeed allowed a precise control of dietary choline and other TMAO precursors. Replacement meals typically provide high-biological-value proteins (15–18 g per serving), minimal fat content (<4 g), and very low available carbohydrates (<3.5 g), enabling strict adherence to ketogenic thresholds [34]. Also, unlike fresh foods, whose nutrient composition may vary considerably, replacement meals reduce uncertainty related to portion size estimation and food composition databases [65,66,67]. Food-based ketogenic diets may include variable amounts of animal-derived products rich in choline and carnitine, while replacement meals provide a standardized nutritional composition, minimizing inter-individual variability in precursor intake since they are mainly formulated with plant protein. This results in improvement in microbiota composition and gut health. As previously reported, a favorable modulation of the *Firmicutes*-to-*Bacteroidetes* ratio was detected in participants adhering to a VLEKT protocol based on meal replacements. On the basis of these findings, the authors inferred that this dietary strategy confers a dual benefit, simultaneously improving metabolic parameters and promoting a more balanced gut microbiota profile [68]. By limiting excessive intake of choline-rich animal foods, thus, replacement meal-based VLEKT may directly contribute to lowering TMAO precursor availability, thereby synergizing with ketosis-induced metabolic adaptations.

An additional aspect that warrants consideration concerns the potential contribution of micronutrients and omega-3 fatty acids provided through the structured supplementation implemented within the protocol. Preclinical evidence and recent clinical studies indicate that vitamins and minerals can significantly modulate both the composition and functional activity of the gut microbiota, influencing the production of microbial metabolites [69,70]. Studies conducted in healthy adults have shown that short-term multivitamin and multimineral interventions can alter microbial community structure and increase short-chain fatty acid production, highlighting a significant interaction between background diet and microbiota responsiveness to supplementation [71]. Similarly, omega-3 fatty acid intake has been reported to exert prebiotic-like effects, promoting the proliferation of beneficial taxa and modulating host inflammatory responses [72,73]. Taken together, these findings suggest that although the reduction in circulating TMAO levels observed in our study is primarily associated with the duration of ketosis exposure and the concomitant reduction in FM, an additional—at least partial—contribution from supplementation cannot be excluded. Such supplementation may have influenced microbial TMAO production and the host metabolic response. Acknowledging this factor underscores the importance of considering integrative nutritional strategies in the modulation of gut-derived metabolites and in the design of protocols that combine dietary interventions with targeted supplementation to maximize metabolic benefits.

A further and clinically relevant aspect of this study is the comprehensive assessment of BC, which allowed discrimination between total weight loss and specific FM reduction. The data clearly indicate that FM loss, rather than body weight reduction alone, is independently associated with TMAO lowering. This finding is clinically relevant, as weight loss achieved through different body compartments may have profoundly different metabolic consequences. Current evidence addressing the relationship between circulating TMAO levels and adiposity remains limited. Experimental data have provided mechanistic insights into this association. In particular, an animal study demonstrated that both antisense oligonucleotide–mediated suppression and genetic ablation of FMO3 conferred protection against high-fat diet–induced obesity in murine models. These findings underscore the involvement of the gut microbiota-derived TMA/FMO3/TMAO axis in driving transcriptional reprogramming within white adipose tissue [74]. Notably, in this study circulating TMAO concentrations were positively correlated with body weight, total FM, mesenteric fat accumulation, and subcutaneous adiposity across multiple inbred mouse strains. Furthermore, in cohorts of individuals with overweight or obesity characterized by metabolic alterations and diverse ethnic backgrounds, hepatic FMO3 expression was directly associated with BMI and waist-to-hip ratio, while showing an inverse relationship with the Matsuda Index, a well-established marker of insulin sensitivity [74]. In agreement with these observations, the results of our previous investigation demonstrate a robust positive association between circulating TMAO concentrations and BMI categories. Therefore, beyond its established role as a biomarker and potential mediator of cardiovascular disease risk, accumulating evidence suggests that microbiota-derived TMAO may represent a relevant environmental contributor to the development of obesity and its related metabolic complications [75]. Furthermore, we also reported a positive association between TMAO levels and visceral adiposity index (VAI) [75]. VAI is a validated surrogate marker of adipocyte dysfunction and has been shown to correlate independently with insulin sensitivity and cardiometabolic risk, even in individuals without obesity in primary care settings [76]. Importantly, VAI has been proposed as a valuable tool for the early identification of insulin resistance and cardiometabolic risk prior to the onset of overt metabolic syndrome [77]. In light of this evidence, the observed association between FM reduction and TMAO reinforces the concept that BC changes are a more meaningful therapeutic target than weight loss per se.

Cumulatively, as elevated circulating TMAO has been consistently associated with increased cardiovascular risk, endothelial dysfunction, and adverse cardiometabolic outcomes [55], the demonstration that a VLEKT can significantly reduce TMAO levels, particularly when sustained over time and accompanied by meaningful FM loss, has relevant clinical implications. These findings suggest that modulation of gut-derived pro-atherogenic metabolites may represent an additional mechanism associated with ketogenic nutritional therapies, beyond weight reduction and glycemic improvement. Similarly, the identification of FM loss and ketosis duration as key predictors of TMAO reduction, supported by regression and ROC analyses, further enhances the translational value of the study, offering potential markers to guide clinical decision-making and treatment monitoring.

Several limitations should be acknowledged. First, the study population consisted exclusively of women, limiting the generalizability of the findings to male subjects. Second, the relatively small sample size may have reduced statistical power for certain analyses and precluded more extensive subgroup evaluations. In addition, the absence of a control group following a ketogenic diet based on conventional choline-rich foods prevents direct comparison between structured replacement meals and food-based ketogenic approaches with potentially different effects on TMAO production. This, however, reinforces the use of properly formulated replacement meals for ketogenic therapy allowing for a more rigorous control of choline and other TMAO precursors, despite their higher price. This highlights the scope of VLEKT as nutritional therapy (not a diet) that, as such, should be based on prescription of specific products [41]. Moreover, gut microbiota composition and functionality were not directly assessed, limiting mechanistic insight into microbiota-mediated TMAO modulation. An additional limitation should be acknowledged. Circulating ketone body concentrations were not quantitatively measured during the intervention; therefore, the presence of nutritional ketosis cannot be formally documented through biochemical parameters. Although the dietary protocol strictly adhered to internationally recognized criteria for very-low-energy ketogenic therapy (i.e., carbohydrate intake < 30 g/day and energy intake < 800 kcal/day), and the observed clinical outcomes are consistent with a ketogenic metabolic adaptation, the absence of direct ketone measurements represents a methodological limitation that should be considered when interpreting the findings. Despite these limitations, this study has several strengths. The use of a well-standardized VLEKT protocol with controlled precursor intake allowed a precise evaluation of dietary effects on TMAO metabolism. BC was comprehensively assessed, enabling differentiation between weight loss and FM reduction, which proved crucial for interpreting metabolic outcomes. The relatively homogeneous population reduced confounding related to sex-specific metabolic differences, allowing clearer interpretation of associations between ketosis duration, FM loss, and TMAO changes. Furthermore, the integration of correlation, regression, and ROC analyses provides a robust and clinically oriented assessment of predictors and thresholds associated with TMAO modulation.

## 5. Conclusions

In conclusion, this study demonstrates that a VLEKT induces a significant reduction in circulating TMAO levels in women with obesity, primarily driven by the duration of ketogenic exposure and FM loss rather than absolute weight reduction. These findings emphasize the need to move beyond a weight-centered paradigm and to consider BC and treatment duration as key therapeutic targets in clinical nutrition. From a translational perspective, the identification of a FM reduction threshold associated with greater TMAO modulation may help refine ketogenic protocols aimed at cardiometabolic risk reduction. Overall, adequately prolonged and structurally controlled ketogenic interventions appear to represent a promising strategy for improving metabolite-driven cardiometabolic profiles in clinical practice.

## Figures and Tables

**Table 1:** Baseline characteristics of study participants.

Parameters (*n* = 43)	Mean	SD
Age (years)	26.84	8.74
Height (cm)	165.86	6.43
Weight (kg)	92.40	10.69
BMI (kg/m^2^)	33.60	3.54
WG (cm)	101.07	11.00
Rz (Ohm)	484.19	52.60
Xc (Ohm)	49.11	6.02
PhA (°)	5.80	0.49
TBW (L)	39.84	3.92
ECW (L)	20.31	2.62
ICW (L)	19.53	1.70
TBW (%)	43.41	4.63
ECW (%)	50.92	0.95
ICW (%)	49.08	0.94
FM (kg)	44.05	9.15
FM (%)	47.33	4.28
SMM (kg)	26.29	3.29
SMM (%)	28.74	4.29
TMAO (µM)	5.11	1.61

Data are presented as mean ± SD. Abbreviations: BMI, body mass index; WG, waist girth; Rz, resistance; Xc, reactance; PhA, phase angle; TBW, total body water; ECW, extracellular body water; ICW, intracellular body water; FM, fat mass; SMM, skeletal muscle mass.

**Table 2:** Pre and post VLEKT variations in study anthropometric and bioelectrical parameters, hydration status, and body compositions variables.

Parameters (*n* = 43)	Post VLEKT (Mean ± SD)	Δ% (Pre/Post VLEKT)	*p*-Value
Weight (kg)	82.02 ± 10.53	−8.04 ± 1.63	**<0.001**
BMI (kg/m^2^)	30.91 ± 3.52	−8.04 ± 1.63	**<0.001**
WG (cm)	93.36 ± 10.23	−7.52 ± 3.54	**<0.001**
Rz (Ohm)	480.42 ± 49.88	−0.63 ± 4.06	0.229
Xc (Ohm)	53.81 ± 6.62	9.90 ± 8.89	**<0.001**
PhA (°)	6.40 ± 0.60	10.54 ± 8.80	**<0.001**
TBW (L)	39.21 ± 4.05	−1.59 ± 2.77	**0.001**
ECW (L)	19.55 ± 2.32	−3.74 ± 3.01	**<0.001**
ICW (L)	19.66 ± 1.78	0.66 ± 3.03	0.175
TBW (%)	46.46 ± 4.97	7.06 ± 3.08	**<0.001**
ECW (%)	49.80 ± 0.98	−2.20 ± 1.19	**<0.001**
ICW (%)	50.20 ± 0.98	2.29 ± 1.26	**<0.001**
FM (kg)	36.556 ± 8.77	−17.34 ± 4.38	**<0.001**
FM (%)	42.57 ± 4.76	−10.17 ± 3.39	**<0.001**
SMM (kg)	26.47 ± 3.23	0.78 ± 3.86	0.269
SMM (%)	31.44 ± 4.54	9.61 ± 4.30	**<0.001**

Data are presented as mean ± SD. Paired-sample Student’s *t*-test was used to compare baseline and post-VLEKT values. A *p*-value in bold denotes a significant difference (*p* < 0.05). ∆% indicates the percent change from baseline to post-VLEKT. Abbreviations: BMI, body mass index; WG, waist girth; Rz, resistance; Xc, reactance; PhA, phase angle; TBW, total body water; ECW, extracellular body water; ICW, intracellular body water; FM, fat mass; SMM, skeletal muscle mass.

**Table 3:** Duration of VLEKT and variations in anthropometric measurement, body composition, and hydration status of study participants above and below the median of variations in circulating TMAO levels.

Parameters	Variation in Circulating TMAO Levels (Δ%)		
	>−41.10% *n* = 23	<−41.10% *n* = 20	*p*-Value
Duration of VLEKT	44.26 ± 3.54	35.65 ± 8.68	**<0.001**
Δ% BMI	−8.55 ± 1.39	−7.44 ± 1.71	**0.028**
Δ% WG	−8.18 ± 1.54	−6.77 ± 4.88	0.228
Δ% Rz	0.22 ± 2.79	−1.61 ± 5.05	0.161
Δ% Xc	11.58 ± 8.89	7.97 ± 8.71	0.188
Δ% PhA	11.56 ± 9.01	9.36 ± 8.62	0.418
Δ% TBW (L)	−2.27 ± 1.78	−0.80 ± 3.47	0.099
Δ% ECW (L)	−4.56 ± 2.13	−2.79 ± 3.60	0.064
Δ% ICW (L)	0.10 ± 2.10	1.30 ± 3.79	0.220
Δ% TBW (%)	6.94 ± 2.35	7.20 ± 3.81	0.794
Δ% ECW (%)	−2.35 ± 1.18	−2.02 ± 1.21	0.367
Δ% ICW (%)	2.43 ± 1.23	2.13 ± 1.31	0.438
Δ% FM (kg)	−18.70 ± 3.84	−15.79 ± 4.52	**0.030**
Δ% FM (%)	−11.14 ± 2.98	−9.07 ± 3.56	**0.047**
Δ% SMM (kg)	−0.36 ± 2.55	1.72 ± 4.87	0.159
Δ% SMM (%)	9.33 ± 3.38	9.92 ± 5.23	0.672

Data are presented as mean ± SD. Paired-sample Student’s *t*-test was used to analyze differences between groups. A *p*-value in bold denotes a significant difference (p < 0.05). ∆% indicates the percent change from baseline to post-VLEKT. Abbreviations: BMI, body mass index; WG, waist girth; Rz, resistance; Xc, reactance; PhA, phase angle; TBW, total body water; ECW, extracellular body water; ICW, intracellular body water; FM, fat mass; SMM, skeletal muscle mass.

**Table 4:** Simple correlations among Δ% TMAO serum levels and changes in the study parameters after VLEKT active phase period.

Parameters	Δ% TMAO	
	r	*p*-Value
Duration of VLEKT (days)	−0.647	**<0.001**
Δ% Weight (kg)	0.403	**0.007**
Δ% BMI (kg/m^2^)	0.403	**0.007**
Δ% WG (cm)	0.118	0.451
Δ% Rz (Ohm)	−0.243	0.116
Δ% Xc (Ohm)	−0.375	**0.013**
Δ% PhA (°)	−0.264	0.088
Δ% TBW (L)	0.299	0.052
Δ% ECW (L)	0.389	**0.010**
Δ% ICW (L)	0.163	0.297
Δ% TBW (%)	0.035	0.823
Δ% ECW (%)	0.300	0.050
Δ% ICW (%)	−0.278	0.071
Δ% FM (kg)	0.427	**0.004**
Δ% FM (%)	0.411	**0.006**
Δ% SMM (kg)	0.238	0.125
Δ% SMM (%)	0.051	0.747

A *p*-value in bold denotes statistical significance (*p* < 0.05). Abbreviations: BMI, body mass index; WG, waist girth; Rz, resistance; Xc, reactance; PhA, phase angle; TBW, total body water; ECW, extracellular body water; ICW, intracellular body water; FM, fat mass; SMM, skeletal muscle mass.

**Table 5:** Multiple regression analysis (stepwise model) with TMAO variations (Δ%) as dependent variable to estimate the prediction value of study parameters.

Parameters	Multiple Regression Analysis			
	*R* ^2^	β	t	*p* Value
**Model 1**	0.405			
Duration of VLEKT		−0.647	−5.440	**<0.001**
Variables excluded: Δ% FM, Δ% BMI, Δ% Xc, Δ% ECW				
**Model 2**	0.656			
Duration of VLEKT		−0.704	−7.731	**<0.001**
Δ% FM		0.506	5.557	**<0.001**
Variables excluded: Δ% BMI, Δ% Xc, Δ% ECW				
**Model 3**	0.691			
Duration of VLEKT		−0.775	−8.476	**<0.001**
Δ% FM		0.962	4.531	**<0.001**
Δ% BMI		−0.496	−2.350	**0.024**
Variables excluded: Δ% Xc, Δ% ECW				

A *p*-value in bold denotes statistical significance (*p* < 0.05). Abbreviations: very-low-energy ketogenic therapy, VLEKT; BMI, body mass index; Xc, reactance; ECW, extracellular body water; FM, fat mass.

## Data Availability

The original contributions presented in the study are included in the article, further inquiries can be directed to the corresponding author.

## Abbreviations

The following abbreviations are used in this manuscript:

BC	Body composition
BIA	Bioelectrical impedance analysis
BMI	Body mass index
CV	Coefficient of variation
CVD	Cardiovascular disease
ECW	Extracellular water
FM	Fat mass
FMO1	Flavin-containing monooxygenase 1
FMO3	Flavin-containing monooxygenase 3
ICW	Intracellular water
LOD	Limits of detection
LOQ	Limits of quantification
QC	Quality control
ROS	Reactive oxygen species
SMM	Skeletal muscle mass
TBW	Total body water
TMA	Trimethylamine
TMAO	Trimethylamine-N-oxide
VAI	Visceral adiposity index
VLEKT	Very-low-energy ketogenic therapy
WG	Waist girth
